# Evaluating AI-Generated Podcasts Versus Traditional Reading for Learning From Medical Articles: Protocol for a Mixed-Design Study Among Resident Physicians

**DOI:** 10.2196/78505

**Published:** 2025-12-12

**Authors:** Matthias Stadler, Constanze Richters, Martin R Fischer, Fabian Hutmacher

**Affiliations:** 1 Institute of Medical Education LMU Klinikum München Germany; 2 Human-Computer-Media Institute Julius-Maximilians-Universität Würzburg Würzburg Germany

**Keywords:** artificial intelligence, AI, podcasts, medical education, cardiology

## Abstract

**Background:**

Podcasts have emerged as a popular medium in medical education over the past decade. Audio learning allows flexibility and may help residents engage with content in new ways. Reading scientific literature is a core skill for residents, yet many residents struggle to comprehend complex research articles. Advances in artificial intelligence (AI) have enabled the automatic generation of podcast-style summaries of documents. It remains unclear whether listening to AI-generated podcast summaries can match the educational value of reading the full text of medical papers, and whether this depends on the complexity of the article.

**Objective:**

This study aims to compare comprehension of medical research papers when learning via an AI-generated audio podcast versus traditional reading. We will examine whether article complexity (narrative vs technical) moderates any difference. We hypothesize an interaction: for a highly complex article, residents who read the full text should achieve a better understanding than those who listen to a summary, whereas for an easier article, the difference between modalities should be smaller.

**Methods:**

We designed a 2×2 mixed factorial study with 60 resident physicians preparing for the board certification in internal medicine or cardiology. All participants will engage with 2 peer-reviewed cardiology articles differing in complexity: a narrative case report on eosinophilic myocarditis and a technical research article on quantifying the vena contracta area using 3-dimensional echocardiography. Each participant will read 1 article and listen to an AI-generated podcast summary of the other, with the order and assignment counterbalanced to control for order effects. The podcasts are created using Google NotebookLM’s experimental *audio overview* feature. Participants will complete a multiple-choice knowledge test for each article. The primary outcomes are comprehension scores for each modality. The secondary outcomes include intrinsic motivation, perceived learning gains, and cognitive load for each condition. Data will be analyzed using a mixed ANOVA to test the main effects of modality and article complexity, as well as their interaction.

**Results:**

Data collection is expected to be completed by early 2026. We will report the trial results according to the CONSORT (Consolidated Standards of Reporting Trials) guidelines, and any deviations from this protocol will be documented and justified. No results are available at the time of publication of this protocol.

**Conclusions:**

This randomized trial will offer evidence on the effectiveness of AI-generated podcast summaries as a learning tool for medical literature. If listening to an AI-generated podcast yields comprehension comparable to or superior to reading the full article, it could validate an innovative, time-saving approach for busy medical trainees. Conversely, if significant deficits are observed in the podcast group (especially for complex content), the findings will highlight the limitations of AI summaries and the continued importance of traditional reading for thorough understanding.

**International Registered Report Identifier (IRRID):**

PRR1-10.2196/78505

## Introduction

Medical education is increasingly embracing digital and asynchronous learning modalities. Podcasts, in particular, have seen a marked rise in their use as supplementary learning tools across many medical specialties. By 2021, an estimated 41% of Americans reported listening to a podcast in the past month [1], and medical education has mirrored this trend with numerous educational podcasts now available for both trainees and practitioners [2,3]. For medical residents, podcasts offer a convenient way to learn on the go and can introduce clinical topics in an engaging, conversational format [4]. The popularity of podcasts suggests that they may help sustain learners’ interest and motivation [5]. However, it is important to evaluate whether learning medical content via podcasts is as effective as more traditional methods.

One traditional learning activity in medical school is reading and appraising journal articles. The ability to read and comprehend scientific papers is fundamental for evidence-based practice and lifelong learning [6]. However, medical residents often find it challenging to digest primary literature, especially when articles are dense or highly technical [7]. Journal club studies have documented that learners struggle to understand complex research papers, sometimes becoming lost in advanced methodologies or jargon [7]. Practical, case-based articles (eg, clinical case reports or case-based discussions) may be easier to follow, as they tend to have concrete patient narratives and applied lessons [8]. In contrast, theoretical or review articles (eg, a detailed review of endocrine pathophysiology) can be abstract, laden with data, and not immediately relatable to clinical practice. This disparity in content complexity could influence how well learners comprehend the material and stay engaged [9].

Beyond accessibility and learner motivation, self-regulation theory [10] predicts that podcasts may offer educational advantages related to repetition, self-pacing, and multitasking [11] if learners are able to use them appropriately. However, using podcasts to access medical journal articles may yield higher cognitive demands than reading the articles. The interplay between cognitive resources and learning outcomes is central to cognitive load theory, which posits that human working memory has a limited capacity crucial for learning [12]. According to cognitive load theory, learning activities typically demand a significant allocation of cognitive resources, and instructional design plays a pivotal role in either exacerbating or alleviating cognitive load. The theory delineates 3 types of cognitive load: extraneous, intrinsic, and germane, each influencing learning in distinct ways [13]. Listening to audio differs cognitively from reading: listeners process information sequentially, at a fixed pace, and may lack easy access to figures or references [14]. In addition, podcasts are inherently reductive; condensing full-text articles into short conversational scripts may reduce extraneous load [15] but risks omitting critical nuance or context [16,17]. This, in turn, may suppress the intrinsic load necessary for deeper understanding, ultimately resulting in shallow learning processes [15]. While learners may perceive these summaries as more enjoyable or efficient, it is unclear whether they support equivalent knowledge acquisition, especially for content of varying complexity.

Producing high-quality medical education podcasts typically requires expert knowledge, pedagogical insight, technical skills, and significant time for scripting, recording, and postproduction [18]. These constraints limit scalability and personalization, especially for institutions or learners aiming to integrate podcasts into dynamic, content-rich environments, such as medical curricula. Recent advances in generative artificial intelligence (AI) present an opportunity to overcome some of these barriers. AI-powered systems based on large language models can now generate podcast-style summaries of academic content by transforming articles into structured, conversational dialogues [16]. These AI-generated podcasts often simulate discussions between 2 virtual hosts, aiming to translate complex material into an engaging audio narrative. Early research indicates that such podcasts can enhance learner motivation and satisfaction, particularly when personalized according to user needs [19]. Some studies suggest comparable or even superior learning outcomes relative to traditional reading in specific contexts [17,19].

Nevertheless, the educational value of these AI-generated summaries remains largely untested in medical education [18]. AI-generated podcasts may lack the necessary accuracy or lose important contexts, which may cause either worse learning outcomes or potentially even misinform learners and endanger patients. Therefore, this study aims to address these questions through a randomized controlled trial comparing learning outcomes across 2 types of medical articles (practical vs theoretical) in the reading and listening modalities. By examining both knowledge acquisition and learners’ motivational and cognitive responses, we aim to assess whether AI-generated podcasts can serve as a viable tool for engaging residents with scientific literature.

The primary objective of this randomized trial is to compare knowledge acquisition from medical journal articles between 2 learning modalities: listening to an AI-generated podcast versus reading the full text. We seek to determine if one modality leads to superior comprehension in medical residents. A secondary objective is to assess the influence of article complexity on learning outcomes and any interaction between complexity and modality.

In operational terms, the study will test two main hypotheses and one interaction hypothesis, as presented in Textbox 1.

**Study hypothesis.**

**Main effect of modality (artificial intelligence [AI]**–**generated podcast vs reading the full text)**

There may be a difference in knowledge scores between residents who listen to an AI-generated audio summary and those who read the article, regardless of complexity. We will explore the direction of this effect; we expect that reading the full article could confer an advantage for understanding, especially detailed content, but well-crafted audio summaries may yield comparable results for certain types of information.

**Main effect of complexity (low vs high)**

We anticipate that all residents, regardless of modality, will find the *complex* article more challenging, resulting in lower average test scores compared to the *easy* article, given the higher theoretical density of the former.

**Modality × complexity interaction**

We hypothesize an interaction such that the effectiveness of the podcast relative to reading will differ by article complexity. Specifically, the performance gap (if any) between listening and reading is expected to be larger for the complex article than for the easy article. In other words, for the easier, case-based article, listening to a summary might achieve nearly the same comprehension as reading, whereas for the complex article, listening to the summary might not capture enough detail, leading to lower comprehension compared to reading the full text.

Additional objectives include evaluating secondary outcomes related to the learning experience: we will compare residents’ intrinsic motivation during the task, subjective cognitive load, and their subjective perception of learning between the 2 modalities. This will provide a more nuanced understanding of how engaging or difficult residents found the learning process in each condition, beyond the factual knowledge gains. Ultimately, the goal is not only to determine which method yields higher test scores, but also to consider which method residents prefer or find more motivating, because an engaging learning experience could encourage more frequent interaction with the scientific literature.

## Methods

All study materials (articles, audio files, and questionnaires) and deidentified data will be made available in an open science repository [20] upon study completion, in line with open science practices.

### Study Design and Setting

We will conduct a single-center, 2-factor mixed experimental study with a 2×2 design. The within-subjects factor is the learning modality (AI-generated podcast vs traditional reading), and the between-subjects factor is the presentation order (ie, which article-modality combination comes first). Each participant will engage with 2 cardiology articles—1 practical case report and 1 technical research paper—experiencing 1 via reading and the other via an AI-generated podcast. The assignment of modality to article and the sequence of presentation will be fully counterbalanced across participants to control for order effects and content bias.

The study will take place at LMU Munich. Each participant will attend a single study session, during which they will read 1 article and listen to the other in podcast form. After each learning task, participants will complete a multiple-choice knowledge test and questionnaires assessing motivation, cognitive load, and perceived learning.

All study sessions will be conducted in a controlled classroom environment at LMU Munich. A researcher will be present throughout the study to supervise the session and ensure adherence to the protocol. The physical setting will be quiet, with individual desks and study materials provided on desktop computers or tablets. Participants in the podcast condition will be provided with noise-canceling headphones to standardize audio delivery.

### Participants and Recruitment

The study population will consist of resident physicians enrolled in specialty training programs at LMU Munich, specifically those preparing for the German board certification (Facharztprüfung) in internal medicine or cardiology. These physicians have completed medical school and are engaged in postgraduate training, making them an ideal population for studying learning strategies that support lifelong engagement with scientific literature.

#### Sample Size Calculation

We conducted an a priori power analysis using G*Power (version 3.1; Heinrich Heine University) [21] for a mixed ANOVA with 1 within-subjects factor (learning modality: podcast vs reading) and 1 between-subjects factor (article-modality order). To detect a moderate effect size (f=0.25) for the main effects or interactions, with α=0.05 and power (1−β=0.80), the required total sample size is approximately N=44. To account for potential dropouts or missing data, we plan to recruit a total sample of N=60 resident physicians.

#### Inclusion Criteria

Participants must be currently enrolled in a medical residency program, specifically in a discipline such as internal medicine or cardiology. Additionally, they must be actively participating in a structured preparation course for the German specialist board certification (Facharztprüfung). Eligible participants should have completed their undergraduate medical education and be in the clinical phase of a postgraduate training.

Because the study materials, including the articles and AI-generated podcast summaries, are presented in English, participants must have sufficient English proficiency to understand both written and spoken academic medical content. While we expect most physicians in the preparation course to possess the necessary language skills, this will be confirmed informally during recruitment.

All participants must be willing and available to participate in a single research session lasting approximately 60 minutes and must provide written informed consent. Furthermore, they must have normal or corrected-to-normal hearing and vision to ensure that they can engage with both the reading and audio conditions, as required by the within-subjects design.

#### Exclusion Criteria

We will exclude individuals who have prior extensive familiarity with the specific articles used in the study. During recruitment and again at the start of the experimental session, we will present the titles and journal references of the 2 articles. If a participant confirms that they have previously read, studied, or discussed either article in detail (eg, in a journal club or as part of examination preparation), they will be thanked and excluded from participation to avoid a knowledge bias.

Additionally, individuals with uncorrected hearing impairment or severe visual impairment that would prevent them from effectively listening to the audio podcast or reading the article will be excluded. Because each participant will engage in both a reading and a listening task, we require all participants to have unimpaired or corrected functional access to both modalities.

We will recruit participants from a structured board examination preparation course organized by LMU Munich. This course attracts physicians actively preparing for their specialty certification and therefore reflects a highly motivated and relevant target group. Participants will be informed about the study through announcements made during the course sessions and follow-up emails. The study will be presented as a research project on learning from medical articles using different formats, without disclosing specific hypotheses. Participation is entirely voluntary and will not affect their standing in the course or the certification process.

Before participation, each physician will receive a full briefing on the study’s goals, procedures, and data privacy protections. This study will generally be described as a comparison of different learning formats for medical content. Written informed consent will be obtained. We will emphasize that participation is voluntary, performance on the comprehension tests has no bearing on their training or certification, and all data will be anonymized and treated confidentially.

### Study Materials

#### Articles (Learning Content)

Two published medical articles have been selected to represent different levels of complexity within the field of cardiology, both published in *BMC Cardiovascular Disorders*.

The *narrative* article is a case-based clinical report [22]. This report describes the diagnostic journey and clinical management of a patient with eosinophilic myocarditis who presented with cardiogenic shock and malignant arrhythmias. The article is structured in a narrative format and emphasizes clinical reasoning, interdisciplinary care, and escalation of treatment. It presents practical, concrete decision-making steps, making it highly accessible and relevant for clinical-phase medical students.

The *technical* article focuses on the validation of 3-dimensional echocardiographic measurements of the vena contracta area for quantifying mitral regurgitation severity [23]. It involves advanced imaging techniques, direct planimetry, multiplanar reconstruction, and statistical comparisons, including sensitivity and specificity analysis and Bland-Altman plots. The dense methodological content and reliance on imaging expertise and statistical interpretation make it cognitively demanding and better suited for advanced learners with prior knowledge of cardiac imaging and valvular pathology.

These articles were deliberately chosen to provide a clear contrast in complexity and content delivery within the same specialty and text length (8 pages), allowing for an effective assessment of learning outcomes across different formats and difficulty levels.

For the reading groups, the article text will be provided on a screen in PDF format. For the podcast groups, the content of these articles is used as input to generate the AI podcasts.

#### AI-Generated Podcasts

Using Google NotebookLM (an AI platform currently in experimental release) [24,25], we generated an audio summary for each of the 2 articles. The process involves uploading the article text into NotebookLM and then using the “audio overview” feature. The output is an audio file featuring 2 synthetic voices (the *AI hosts*) discussing the article. According to NotebookLM’s design, the hosts begin by introducing the topic, then proceed to discuss key points, clarify terms, and make connections. This is similar to one host quizzing or interviewing the other about the article’s content. The tone is meant to be conversational, which can help maintain listener interest.

Google NotebookLM was chosen for this study because, at the time of design, it was the only publicly accessible platform capable of generating conversational, dialogue-style audio summaries directly from full-text academic articles with no need for additional scripting. Its *audio overview* feature automatically organizes content into a simulated exchange between 2 hosts. This format aligns with well-established educational strategies, such as elaborative interrogation and peer explanation, which can foster learner engagement and retention. While alternative AI tools can produce summaries, they typically provide plain-text outputs or monologue-style narrations and require substantial manual adaptation to achieve a similar conversational format. Using NotebookLM enabled us to create standardized, pedagogically relevant podcasts with minimal human editing, thereby reducing the risk of inadvertent bias in the intervention materials while maintaining ecological validity for real-world use cases.

Each resulting podcast is approximately 15 minutes in length. The audio files are in English, matching the article's language. We reviewed the transcripts of these AI-generated discussions to ensure they cover the main sections of the articles (eg, the case presentation and outcome in the narrative article, and all major subtopics in the technical article). We also checked for any factual inaccuracies or misrepresentations.

During the session, participants in the podcast conditions will listen to the audio using over-ear headphones provided by the study team (to ensure good sound quality and noise isolation). The audio will be played on a tablet or computer via a simple media player interface. Participants will be instructed that they can pause, rewind, or replay the audio as needed (up to 1 full listening of the entire length again, if time allows), but they cannot fast-forward beyond sections they have not heard yet (to prevent skipping content). They will not have a transcript or any text of the article while listening to simulate a true audio-only learning experience.

#### English Proficiency

Participants will rate their level of English proficiency along the dimensions of *listening*, *reading*, *participating in conversations*, and *coherent speech* [26]. For each dimension, they will be given 6 levels (from A1 to C2) with short descriptions, such as “I can understand individual words and parts of sentences” (listening A1) or “I can write clear, fluent, stylistically appropriate reports, articles, and sophisticated letters” (coherent speech C2). English proficiency will be considered sufficient if no dimension is reported to be below B2.

#### Knowledge Tests

For each article, we developed a multiple-choice questionnaire to assess the comprehension and retention of key information. Each test consists of 10 questions in a single-best-answer format, and the sum of all correct answers represents the knowledge score. The questions cover important points from the article.

All questions were written specifically for this study. We ensured that each question could be answered based on the information in the article and that the AI podcast, if accurate, would also cover those points. We had 2 faculty members with a background in cardiology who were not involved in the study review the questions for content validity and clarity. Internal testing of the final items yielded no problems with comprehensibility.

#### Motivation

To gauge participants’ motivation and engagement during the learning task, we will use items from the Intrinsic Motivation Inventory (IMI) [27], a well-established instrument for assessing subjective motivation in experimental tasks. We will focus on the interest and enjoyment as well as the effort and importance subscales of the IMI, as these are most relevant here. The interest and enjoyment scale serves as a measure of how enjoyable or engaging the student found the activity (considered a self-report proxy for intrinsic motivation), whereas effort and importance indicate how much effort they invested and how important they perceived doing well to be. Participants rate their agreement with the scales’ 7 (interest/enjoyment) and 6 (effort/importance) statements (eg, “I enjoyed doing this activity very much”; “I put a lot of effort into this”) on a 7-point Likert response from “strongly disagree” to “strongly agree.” The IMI has been used in educational studies and has shown good psychometric properties in various contexts (Cronbach α >0.80 in similar studies) [28], allowing the use of mean scores across all items of a scale as the final score.

#### Cognitive Load

We will assess the cognitive load experienced by participants using the questionnaire developed by Klepsch et al [29]. This instrument allows for a separate evaluation of intrinsic load (the inherent difficulty of the content), extraneous load (the load imposed by the way information is presented), and germane load (the effort invested in meaningful learning). We will use the version with a total of 8 statements rated on a 7-point Likert response (“not at all true” to “completely true”). Examples of items include “The content was very complex” (intrinsic load), “The way the information was presented was confusing” (extraneous load), and “I invested a lot of effort into understanding the material” (germane load). All 3 scales showed good internal consistencies (Cronbach α >0.80 in similar studies), allowing the use of mean scores across all items of a scale as the final score.

#### Perceived Learning

We will ask participants to self-assess their perceived learning from the activity. This will be done with 3 custom questions adapted from prior studies on perceived learning [30]. Responses will indicate agreement on a 7-point Likert response from “strongly disagree” to “strongly agree.” The 3 questions are “Overall, I was satisfied with my learning experience,” “This type of learning met my needs as a learner,” and “I could learn well with the material presented.”

### Procedure

Each participant will attend a single session, either individually or in small groups, depending on laboratory capacity. Even when in groups, each participant will complete the study independently at their own workstation (with individual screens and headphones).

#### Orientation (5 Min)

Participants are welcomed and seated at a computer or tablet station. Written informed consent is obtained if not already collected during recruitment. Participants then complete a short intake form to gather demographic information (age, gender, and specialty area), confirm their participation in the Facharztprüfung preparation course, and verify their English proficiency and lack of prior exposure to the study materials. They are also screened for any sensory impairments that might interfere with reading or listening tasks.

Participants are randomly assigned to one of the counterbalanced order conditions (ie, whether they first read the narrative article or listen to the technical podcast, or vice versa). The researcher then provides access to the appropriate learning material.

#### Learning Phase 1 (30 Min)

Participants begin with their first learning task: either reading 1 article or listening to the podcast version of the other. Each participant has up to 30 minutes for this phase, which accommodates the full podcast duration (approximately 12 min) or sufficient reading time (approximately 15 min) with sufficient buffer. Participants are instructed not to take notes or use external aids. The researcher monitors to ensure compliance (eg, that readers are not using mobile phones or writing and that listeners are not replaying segments excessively).

#### Knowledge Test 1 (5 Min)

Immediately after the first learning task, the materials are removed. Participants complete a multiple-choice quiz assessing their comprehension of the content they just engaged with.

#### Posttask Questionnaire 1 (5 Min)

Participants complete a brief questionnaire assessing intrinsic motivation, cognitive load, and perceived learning for the first learning task.

#### Break (Optional, Approximately 2 Min)

A short, optional break (2-3 min) may be offered to reduce carryover fatigue between tasks.

#### Learning Phase 2 (30 Min)

Participants proceed to their second learning task, which is in the opposite modality and with the alternate article. Again, materials are provided for up to 30 minutes, and participants are instructed not to take notes or access external resources. The researcher continues to monitor the session.

#### Knowledge Test 2 (5 Min)

As before, the article or podcast is removed, and participants complete a second multiple-choice quiz focused on the second learning task.

#### Posttask Questionnaire 2 (5 Min)

Participants complete the same set of questions (motivation, cognitive load, and perceived learning) for the second task.

#### Debriefing (5 Min)

After all tasks are complete, the researcher provides a brief debriefing. Participants are informed about the study’s purpose and hypotheses and may ask questions. If they are interested, they are offered access to the alternate format for each article (eg, if they read the case report, they may request the podcast version, and vice versa). Participants are politely asked not to discuss the study specifics with peers until the study has concluded to minimize bias for future participants.

### Data Analysis Plan

#### Descriptive Statistics

Only cases with complete data will be used, with no imputations of missing values. We will summarize participant demographics (age, gender, specialty training area, and prior exposure to similar materials) for the entire sample and by order group (eg, whether the participant read first or listened first). We will report the mean and SD for all primary and secondary outcome measures (knowledge test scores, motivation, perceived learning, and cognitive load) by modality and article complexity.

#### Primary Analysis

To assess the effect of learning modality and article complexity on comprehension, we will conduct a 2×2 mixed ANOVA with the following factors:

- Within-subjects factor: modality (AI-generated podcast vs traditional reading)
- Within-subjects factor: article complexity (narrative case report vs technical research article)

Each participant completes both a reading task and a podcast task, 1 with the narrative article and the other with the technical article. The assignment of modality to article is counterbalanced. We will test for the main effects of modality and complexity, and their interaction, on knowledge test scores (range 0-10 per article). Effect sizes will be reported using eta squared (η²). Assumptions of normality (Shapiro-Wilk test on residuals) and homogeneity of variance (Levene test) will be examined. If assumption violations are detected, we will consider using robust ANOVA procedures [31].

#### Secondary Analyses

We will perform similar 2×2 mixed ANOVAs for each of the following secondary outcomes:

- Intrinsic motivation, measured using the interest and enjoyment subscale of the IMI
- Cognitive load, using the scale developed by Klepsch et al [29], which includes subscales for intrinsic, extraneous, and germane load
- Perceived learning gain, rated on a single-item Likert scale

Each participant completes these measures after each learning task, allowing within-subjects comparisons across modalities and content types.

For all analyses, we will consider both the effect size and the *P* value when interpreting the results. To document test quality, we will report psychometric properties (eg, internal consistency and item characteristics) for both multiple-choice questionnaires. Differences in mean scores are expected due to content difficulty, but reliability (McDonald’s Omega) will be used to ensure comparability across tests.

### Ethical Considerations

This study was approved by the Ethics Committee of LMU Munich (23-0688). All procedures were reviewed for compliance with ethical standards in research with human participants.

#### Informed Consent

As noted, we obtain written informed consent from all participants. They are informed that the study is about learning from the medical literature and involves completing tests, but the full details of the interventions are somewhat masked until debriefing to preserve the blinding of the hypothesis. They are told that they can withdraw at any time without penalty.

#### Confidentiality

Each participant is assigned a code; no identifiable personal information is associated with their data. Any publications or reports will present only aggregated results. We do not foresee collecting sensitive personal information; the demographic data collected is minimal.

#### Compensation

As an incentive, all participants will be given the option to enter a lottery to win a €50 (US $58) gift voucher.

## Results

As of the submission of this protocol (August 2025), no participants have been recruited, and no data have been collected. Once data analysis is complete (by early 2026), we will report the trial results according to the CONSORT (Consolidated Standards of Reporting Trials) guidelines, and any deviations from this protocol will be documented and justified. No results are available at the time of publication of this protocol.

## Discussion

### Anticipated Findings

This study evaluates AI-generated audio summaries (*AI podcasts*) as a tool for learning from scientific literature in postgraduate medical education. By directly comparing podcast-based learning with the traditional method of reading full-text articles, we aim to generate evidence on comprehension outcomes, learner motivation, cognitive load, and perceived learning. Because each participant will experience both modalities—reading and listening—our within-subjects design allows for more precise comparisons while controlling for individual differences in prior knowledge, attention, and learning preferences.

The findings will have practical implications for adapting learning materials for resident physicians preparing for board certification. If we find that reading leads to significantly better comprehension than listening, particularly for complex or technical content, this suggests that caution is warranted in using audio summaries as a standalone resource. Resident physicians may miss subtle but clinically important nuances when relying solely on AI-generated podcasts for learning. In such a case, AI-generated audio could still be valuable as a supplementary tool—useful for introducing new material, reinforcing key ideas, or supporting review during time-constrained situations such as commuting or night shifts. The results could also inform developers and content curators about the limitations of current AI summarization models, especially for dense scientific material, and point to areas where greater fidelity and detail are needed.

On the other hand, if comprehension is equivalent across modalities, even for technical articles, this would support the integration of AI-generated audio into formal and informal learning routines. AI podcasts could then be leveraged to increase the flexibility with which physicians access and absorb scientific information, without compromising accuracy or depth. Medical educators and institutions might consider systematically producing and offering AI-generated audio summaries of core readings or recent guideline updates, especially for busy clinicians with limited time for traditional study methods.

Beyond comprehension, our secondary outcomes will help evaluate learner motivation and cognitive engagement. If AI podcasts are associated with higher intrinsic motivation or perceived enjoyment, this could be particularly relevant in postgraduate medical training, where learners face high workloads, cognitive fatigue, and declining study motivation. A more engaging modality could contribute to improved study adherence or knowledge retention over time. Even if comprehension is slightly lower in the podcast format, enhanced motivation might still justify its use—especially as a tool for sustained exposure or re-engagement with difficult topics. Conversely, if resident physicians report lower interest or higher cognitive load when listening, this will provide important feedback for educators and tool developers alike.

### Limitations

We acknowledge several limitations of our study. First, our findings will be specific to the 2 articles chosen. While we intentionally selected examples of narrative and technical articles, they are only single instances of those categories. Thus, generalizing to all *narrative vs technical* content should be done cautiously. The results might differ with other topics or types of papers. Ideally, a follow-up study with more varied content would strengthen generalizability. Moreover, the expert screening of AI-generated podcasts may be influenced by confirmation bias during transcript editing, suggesting the need for additional studies replicating our findings based on different podcast outputs. Moreover, article identity is confounded with complexity, as only 1 narrative and 1 technical paper were used. This limits external validity and precludes treating article as a random factor. Future studies should include multiple articles per complexity level to strengthen generalizability and allow for modeling article-level variance.

Furthermore, our design does not fully disentangle the effects of learning modality (listening vs reading) from those of content completeness (summary vs full text). The AI-generated podcasts used in this study provide condensed, dialogue-style overviews and do not provide access to the original figures or data tables. In contrast, the reading condition involves the complete article. Consequently, any differences in comprehension could reflect the presentation modality and informational scope. We intentionally opted for this naturalistic comparison because AI-generated summaries are likely to be used as practical alternatives to reading full articles in real-world contexts. Nevertheless, future research should seek to match content more closely across modalities, for instance, by comparing narrated full-text versions with written full texts, or written summaries with audio summaries, and ensuring equitable access to visuals. Such studies would help clarify whether comprehension differences stem primarily from modality or from differences in content coverage and depth.

Finally, our sample of resident physicians preparing for board certification at a single academic medical center in Germany may not be representative of all medical learners. While many of these participants are accustomed to engaging with English-language scientific content and are relatively comfortable with digital tools, their exposure to audio-based learning formats, such as podcasts, varies. The results may differ in other training environments or cultural contexts, particularly where English proficiency is more variable or digital learning is less embedded. The major advantage of this sample lies in its ecological validity and the participants’ strong background in internal medicine. This limits the potential variation due to differences in previous knowledge.

### Conclusions

In conclusion, the forthcoming results of our study will help determine whether AI-generated podcast summaries can serve as an effective study method for medical residents and how content complexity influences this. If successful, this could open the door to new learning strategies and tools in medical education, making the vast realm of medical literature more accessible through AI assistance. If challenges are identified, our work will highlight what needs to be addressed, whether improving AI summarization fidelity or guiding students on when to use such tools. Ultimately, this study aligns with the goal of leveraging technology to enhance learning while rigorously testing its efficacy and ensuring that educational innovations are evidence-based.

## Abbreviations

AI: artificial intelligence
CONSORT: Consolidated Standards of Reporting Trials
IMI: Intrinsic Motivation Inventory

## Data Availability

The datasets generated or analyzed during this study are available in the Open Science Framework repository [20].
